# Supplementary material for: Automated discovery of a robust interatomic potential for aluminum
Source: Nat Commun. 2021 Feb 23;12:1257. doi: 10.1038/s41467-021-21376-0 (PMC7902823; doi:10.1038/s41467-021-21376-0)
Supplement: Supplementary file 1 — Supplementary Information [file 41467_2021_21376_MOESM1_ESM.pdf]

Supplemental Material for: *"Automated discovery of a robust interatomic potential for aluminum"*

Justin S. Smith*[1,2], Benjamin Nebgen†[1], Nithin Mathew[1,2], Jie Chen[3], Nicholas Lubbers[4], Leonid Burakovsky[1], Sergei Tretiak[1], Hai Ah Nam[4], Timothy Germann[1], Saryu Fensin[3] and Kipton Barros‡[1]

[1]Theoretical Division, Los Alamos National Laboratory, Los Alamos, NM
[2]Center for Nonlinear Studies, Los Alamos National Laboratory, Los Alamos, NM
[3]Materials Division, Los Alamos National Laboratory, Los Alamos, NM
[4]Computer, Computational, and Statistical Sciences Division, Los Alamos National Laboratory, Los Alamos, NM

Supplementary Note 1 DFT Calculations

We used the Quantum Espresso [1, 2] software package, using a 3 electron PBE ultrasoft pseudopotential (*Al.pbe-n-rrkjus_psl.0.1.UPF*). All calculations we performed using a kinetic energy cutoff of 20 Ry and a $3 \times 3 \times 3$ k-point grid. In retrospect, a larger basis would have been more appropriate for the linear dimensions of our supercells, ranging from 10.5 Å to 17.0 Å (units cells contain up to 250 atoms). We used the Marzari-Vanderbilt [3] method of thermal electron smearing with an electronic temperature of 0.06 Ry. The SCF iterations were converged to within 10^{-8} Ry. Each Quantum Espresso calculation was parallelized over four Nvidia V100 GPUs; the typical wall clock time for a QE calculation with 124 atoms was 8 minutes.

Supplementary Note 2 Active Learning Details

To bootstrap the active learning procedure, we generate an initial data set of 401 DFT calculations, performed on randomized atomic configurations. Each such configuration contains between 55 to 249 Al atoms, randomly placed in an orthorhombic supercell, subject to a non-overlapping constraint, as described in the main text. Active learning proceeds in iterations. At each iteration, ANI neural networks are trained on all available DFT data, and used to carry out molecular dynamics (MD) simulations for the purpose of sampling. We use an MD step size of 1 fs. MD initial conditions are randomized. To diversify the sampling, each MD run is driven according to a randomized heating and cooling schedule as described in the main text. An uncertainty quantification technique is utilized to determine whether the ANI-Al model (under construction) is confident in its predictions for the current configuration of atoms. To estimate model uncertainty, we employ the ensemble disagreement technique used in previous work [4]. Specifically, we look at the sample

*just@lanl.gov
†bnebgen@lanl.gov
‡kbarros@lanl.gov

variance $\mathrm{Var}[\hat{E}]$ of the energy predictions $\hat{E}$ over the eight neural nets that comprise a single (ensembled) ANI-Al model. We also look at the ensemble variance for the force predictions, $\mathrm{Var}[\hat{\mathbf{f}}]$, averaged over all N atoms in the unit cell. If, during active learning, the ensemble variance for energy or force prediction exceeds a threshold ($E_{\text{thresh}} = 0.001\sqrt{N}\,\text{eV}$ or $f_{\text{thresh}} = 0.002\,\text{eV/Å}$, respectively) then a new DFT calculation is queued to help the ML model to better understand that atomic configuration. If an uncertainty threshold is exceeded, we halt MD simulation and queue a DFT calculation to acquire ground-truth training data for the current MD configuration. If an MD simulation reaches 8 hours of wall-clock time (about 200ps to 500ps of simulated time) without reaching the uncertainty threshold, it is respawned. Batches of MD simulations are carried out with multiple ANI-Al models simultaneously. DFT calculations are continuously performed on the queued configurations. If 2 hours pass since the last ANI-Al model was trained and more than 25 new data points had been generated, a new ANI-Al model is trained. In this way, full cycles of ensemble training, MD simulations for sampling, and DFT data generation are carried out simultaneously to build the training data set. Our final training data set contains 6,352 DFT calculations. Each DFT calculation provides considerable data: a supercell contains between 55 and 249 Al atoms, and DFT calculates forces for each atom.

Figure 7 below illustrates the workflow for the MD simulations used during active learning.

Supplementary Note 3 ANI-Al Model Hyper-parameters

The ANI neural networks used in this work were implemented in the NeuroChem C++/CUDA software package. A batch size of 128 was used while training the ANI-Al model. A weight of 1.0 was used on the energy loss term of the loss function, while a weight of 0.01 was used on the force training loss term. Learning rate annealing was used during training, starting at a learning rate of 0.001 and converging at a learning rate of 0.00001. The learning rate annealing algorithm is described in previous work [5]. The ADAM [6] update algorithm is used during training. The network architecture is provided in Table 1.

All ANI-Al model symmetry function parameters are provided below:
Radial Cutoff (Radial): 7.0
Radial Cutoff (Angular): 5.0
Radial Eta: [43.9]
Radial Shift: [1.2500000,1.4296875,1.6093750e,1.7890625,1.9687500e,
2.1484375,2.3281250,2.5078125,2.6875000,2.8671875,3.0468750,
3.2265625,3.4062500,3.5859375,3.7656250,3.9453125,4.1250000,
4.3046875,4.4843750,4.6640625,4.8437500,5.0234375,5.2031250,
5.3828125,5.5625000,5.7421875,5.9218750,6.1015625,6.2812500,
6.4609375,6.6406250,6.8203125]
Angular Zeta: [69.4]
Angular Angular Shift: [0.19634954,0.58904862,0.98174770,1.3744468,1.7671459e,
2.1598449,2.5525440,2.9452431]
Angular Eta: [6.5]
Angular Radial Shift: [1.2500000,1.7187500,2.1875000,2.6562500,3.1250000,
3.5937500,4.0625000,4.5312500]

Supplementary Note 4 Nearsightedness principle and the interaction cutoff distance

The ANI-Al predicted total system energy $\hat{E} = \sum_i \hat{E}_i$ is a sum over local contributions centered on each atom i. For the hyperparameters listed above, each local energy $\hat{E}_i$ incorporates information about neighboring atoms j if the pairwise distance $r_{ij} = |\mathbf{r}_i - \mathbf{r}_j|$ is less than 7 Å. Furthermore, $\hat{E}_i$ can incorporate angular information $\mathbf{r}_{ij} \cdot \mathbf{r}_{ik}$ involving neighboring atoms j and k if *both* are within a smaller cutoff distance: $r_{ij} < 5$ Å and $r_{ik} < 5$ Å. Using i as an intermediary, the total ANI-Al energy can strongly couple atoms j and k separated by a distance of up to 10 Å, even though the "cutoff distance" is just 7 Å

The ANI-Al predicted force on atom j is $\mathbf{f}_j = -\mathrm{d}\hat{E}/\mathrm{d}\mathbf{r}_j$. Note that $\mathbf{f}_j$ involves $-\mathrm{d}\hat{E}_i/\mathrm{d}\mathbf{r}_j$ if $r_{ij} < 7$ Å. In turn, $\hat{E}_i$ can depend on atom k if $r_{ik} < 7$ Å. Considering a linear geometry in which atoms j, i, and k are separated by 7 Å intervals, one observes that $\mathbf{f}_j$ may in principle be affected by an atom k for pairwise distances of up to $r_{jk} < 14$ Å.

Supplementary Note 5 Training to forces

We employed the loss function

$$L = \left(\hat{E} - E\right)^2 + \ell_0^2 \sum_{j=1}^{N} \left(\hat{\mathbf{f}}_j - \mathbf{f}_j\right)^2, \tag{1}$$

summed over systems in the dataset.

Stochastic gradient descent training requires calculation of all components $\partial L/\partial W_i$ of the loss gradient. Typically, there are order 10^5 model parameters W_i.

Frameworks such as TensorFlow or PyTorch support iterated backpropagation, thereby enabling efficient calculation of the full gradient $\partial L/\partial W_i$ at a cost comparable to calculating L itself [7, 8, 9].

For the present work, we used the C++ Neurochem implementation of ANI [10], for which iterated backpropagation would be challenging to implement. Instead, we use a recently developed

scheme to train to force data [11]. The force part of the loss gradient $\partial L/\partial W_i$ can be cast as a *directional derivative* of $\partial \hat{E}_i[\mathbf{r}]/\partial W_i$, in which each atomic position $\mathbf{r}_j$ is varied in the direction of the observed force prediction error. Using central differences, the loss gradient may be approximated as

$$\frac{\partial L}{\partial W_i} = 2\left(\hat{E} - E\right)\frac{\partial \hat{E}}{\partial W_i} - \frac{\ell_0^2}{\eta}\left(\frac{\partial \hat{E}[\mathbf{r}^+]}{\partial W_i} - \frac{\partial \hat{E}[\mathbf{r}^-]}{\partial W_i}\right)^2, \tag{2}$$

where $\mathbf{r}_j^\pm = \mathbf{r}_j \pm \eta(\hat{\mathbf{f}}_j - \mathbf{f}_j)$ represents a carefully selected perturbation to the position of atom j (for all j simultaneously). Crucially, $\mathbf{r}_j^\pm$ is treated as fixed with respect to variations in model parameters W_i. This central difference approximation would be exact in the limit that $\eta \to 0$. Guidance on selecting η at fixed floating point precision is provided in Ref. [11]. This scheme is highly efficient because, for a given atomic configuration, the full energy gradient $\partial \hat{E}/\partial W_i$, for all model parameters W_i, can be efficiently calculated using straightforward backpropagation (i.e., using the same techniques as needed to implement energy-only training). In practice, we find that the cost to calculate all $\partial L/\partial W_i$ is of the same order as the cost to calculate all forces.

Supplementary Note 6 t-SNE Embeddings

For each atom in the active learning generated data set we obtain a chemical environment descriptor, which is the first hidden layer of an ANI-Al model. These chemical environment descriptors are embedded into a 2D space using the OpenTSNE python package [12]. The t-SNE algorithm is initialized with principal component analysis (PCA). The cosine distance metric is used with a perplexity of 100.

Supplementary Note 7 MD Simulation Details

Melting temperatures via solid-liquid coexistence. The melt-curve was determined using solid-liquid coexistence simulations [13, 14, 15] performed in the NPT ensemble. Independent NPT simulations were performed to determine equilibrium densities of solid/liquid Al determined at the relevant (P,T) conditions. The starting configurations for solid-liquid coexistence simulations, containing 16000 atoms, were created using these equilibrium densities. The simulation cells were equilibrated in NVT ensemble for 100 ps, at a temperature close to the expected melting point. At the end of the equilibration, the simulation cell consisted of liquid Al in contact with the (100) crystallographic face of Al, with roughly equal number of atoms in the solid and liquid phases. Following this, independent NPT simulations of solid-liquid coexistence were performed at a set of temperatures spanning the melting temperature at a given pressure. The time evolution of the simulation-cell volume was used to determine the upper and lower bounds of the melting temperature, i.e., an increase/decrease in the simulation cell volume with time implied that the system was above/below the melting temperature. All the melting-points presented in Figure 6 of the main article are the upper bounds determined as explained. Lower bounds are at the most 25 K lower than the reported upper bounds.

We note that a fcc-bcc phase transition was predicted for the Mendelev-EAM potential [16] at P > 20 GPa. Therefore, we calculated the melt-curve for this potential only up to 15 GPa.

Phase Transition Dynamics. For this simulation, we first slowly heat the system from 300 K to 1500 K, passing through the melt point of 933 K at atmospheric pressure. Next we slowly cool the system back to 300 K. The entire heating-cooling process runs for 750 ps. As with our previous MD simulations, we use a Langevin thermostat with a friction coefficient of 0.02 fs^{-1}, and we apply

an MD timestep of 0.5 fs. The simulation is performed in a periodic box containing 108 atoms initialized in an FCC lattice (that is, $3 \times 3 \times 3$ cubic unit cells). The density of the simulation is fixed by selecting a volume of $(12.138\,\text{Å})^3$. At this density, the simulated pressure should be about 1 bar at the coexistence temperature.

Variable temperature and density liquid phase simulations. We iterated through five temperatures, 1000 K, 1200 K, 1400 K, 1600 K, and 1800 K. At each temperature, we used ANI-Al to drive MD simulations of 108 atoms at five volumes, $0.850V_0$, $0.925V_0$, $1.00V_0$, $1.075V_0$, and $1.15V_0$, where V_0 is the mean volume at zero pressure. Each simulation was carried out for 60 ps. We sampled 10 snapshots from each of the 5×5 MD trajectories. Figure S5 shows the accuracy of the ANI-Al model on each trajectory.

Supplementary Table 1: ANI-Al active learning trained model architecture.

Layer ID	Nodes	Activation
1	96	CELU
2	96	CELU
3	64	CELU
4	1	Linear

Supplementary Table 2: Holdout test set performance for ANI and HIP-NN models on the Al data set. These are single model, not ensemble errors.

Property	ANI	HIP-NN
Energy RMSE (meV/atom)	2.64	3.45
Force RMSE (eV/Å)	0.067	0.130

Supplementary Table 3: Various calculated/experimental properties for FCC aluminum: lattice constant a, bulk modulus B, elastic constants C, energy of formation for vacancy (E^f_{vac}) and $\langle 100 \rangle$ dumbbell interstitial (E^f_{inter}) defects, surface energy E_{surf} (experimental data is averaged over orientations), surface relaxation the between the two outermost layers (d_{12}) and just below that (d_{23}), unstable stacking fault energy E_{usf}, stacking fault energy E_{sf}, unstable twinning fault energy E_{utf}, and melting temperature at atmospheric pressure T_{melt}.

Property	Li04	Me08	Mi99	Zo03	Sh10	Wi09	Pa15	ANI	DFT	Expt.
a (Å)	4.032	4.045	4.050	4.050	4.018	4.025	4.050	4.054	4.042	4.046 ± 0.004 [17]
B (GPa)	81.6	75.1	79.0	79.0	82.6	79.1	78.9	77.3	76.7	79.0 [18]
C_{11} (GPa)	119	106	114	117	119	114	114	117	106	114 [18]
C_{12} (GPa)	62.9	59.5	61.6	60.1	64.1	61.7	61.6	57.2	62.3	61.9 [18]
C_{44} (GPa)	32.9	30.7	31.6	31.7	30.8	31.3	45.4	30.4	31.6	31.6 [18]
E^f_{vac} (meV)	683	659	676	709	673	664	671	663	618	680 [19], 660 ± 20 [20]
E^f_{inter} (eV)	2.72	2.34	2.60	2.21	2.29	2.61	3.03	2.49	2.85	-
E^{100}_{surf} ($\frac{\mathrm{mJ}}{\mathrm{m}^2}$)	1002	496	944	607	842	912	977	990	929	902 ± 78 [21], 980 [22],
E^{110}_{surf} ($\frac{\mathrm{mJ}}{\mathrm{m}^2}$)	1107	581	1006	793	916	1047	1123	1015	997	902 ± 78 [21], 980 [22],
E^{111}_{surf} ($\frac{\mathrm{mJ}}{\mathrm{m}^2}$)	912	428	871	601	813	876	718	855	827	902 ± 78 [21], 980 [22],
d^{100}_{12} (%)	-2.22	-4.15	-0.57	-5.89	-1.93	-0.41	10.54	-1.02	1.21	2.0 ± 0.8 [23]
d^{100}_{23} (%)	-1.69	-0.84	-0.92	-4.15	-1.69	-0.73	2.54	0.69	0.2	1.2 ± 0.7 [23]
d^{110}_{12} (%)	-4.09	-7.66	-1.53	-9.10	-3.20	-3.01	8.20	-10.63	-8.11	-8.5 ± 1.0 [24]
d^{110}_{23} (%)	1.85	-0.34	1.53	-1.85	0.41	1.17	6.33	6.91	4.34	5.5 ± 1.0 [24]
d^{111}_{12} (%)	0.36	-3.66	0.02	-4.49	-1.15	-0.30	7.90	0.7	1.27	0.9 ± 0.7 [25]
d^{111}_{23} (%)	-0.21	-0.12	-0.30	-1.71	-0.91	-0.05	2.21	0.03	0.22	–
E_{usf} ($\frac{\mathrm{mJ}}{\mathrm{m}^2}$)	163	220	168	150	143	180	307	181	179	-
E_{sf} ($\frac{\mathrm{mJ}}{\mathrm{m}^2}$)	131	127	146	114	117	141	187	144	159	166 [22], 120 [26]
E_{utf} ($\frac{\mathrm{mJ}}{\mathrm{m}^2}$)	204	276	220	198	181	234	350	237	254	-
T_{melt} (K)	930	925	1050	875	740	850	950	925	-	933 [27]

Supplementary Table 4: Comparison of FCC property predictions for three ML potentials of aluminum: Physically Informed Neural Network (PINN) [28], DeepPot (DP) [29], and the present work (ANI-Al). Percent errors are given with respect to the reference DFT calculations original reported for each model.

	PINN DFT	PINN	DP DFT	DP	ANI-Al DFT	ANI-Al	PINN Error	DP Error	ANI-Al Error
a (Å)	4.039	4.0396	4.0396	4.0421	4.042	4.054	**0.01%**	0.06%	0.30%
B (GPa)	83	79	78	80.1	76.7	77.3	-4.82%	2.69%	**0.78%**
C_{11} (GPa)	104	117	111	120.9	106	117	12.50%	**8.92%**	10.38%
C_{12} (GPa)	73	60	61.4	59.6	62.3	57.2	-17.81%	**-2.93%**	-8.19%
C_{44} (GPa)	32	32	36.8	40.4	31.6	30.41	**0.00%**	9.78%	-3.77%
E_{surf}^{100} ($\frac{\text{mJ}}{\text{m}^2}$)	920	899	920	950	929	990	**-2.28%**	3.26%	6.57%
E_{surf}^{110} ($\frac{\text{mJ}}{\text{m}^2}$)	980	952	980	990	997	1015	-2.86%	**1.02%**	1.81%
E_{surf}^{111} ($\frac{\text{mJ}}{\text{m}^2}$)	800	819	800	820	827	855	**2.38%**	2.50%	3.39%
E_{vac}^{f} (meV)	665	678	670	790	618	663	**1.95%**	17.91%	7.28%
E_{inter}^{f} (eV)	2.607	2.246	-	-	2.85	2.49	-13.85%	-	**-12.63%**
E_{sf} ($\frac{\text{mJ}}{\text{m}^2}$)	158	121	142	132	159	144	-23.42%	**-7.04%**	-9.43%
E_{usf} ($\frac{\text{mJ}}{\text{m}^2}$)	175	132	-	-	179	181	-24.57%	-	**1.12%**

Supplementary Table 5: Theoretically predicted crystal energies, relative to the ground state. Crystals included are: diamond cubic, simple cubic, A5, $L1_2$ (face-centered cubic with one vacant site), body-centered cubic, A15, hexagonal close packed, 9R, double hexagonal close packed, and face-centered cubic. Units are eV/atom.

Crystal	Li04	Me08	Mi99	Zo03	Sh10	Wi09	Pa15	ANI-Al	DFT
DC	0.8879	0.8123	0.8909	0.8842	0.7006	1.0388	1.0633	0.6788	0.7333
SC	0.3557	0.3124	0.3986	0.2966	0.2298	0.2208	0.39	0.386	0.3695
A5 (β-Sn)	0.2966	0.2664	0.3241	0.2182	0.1752	0.1906	0.2357	0.2592	0.2722
$L1_2$	0.2621	0.2863	0.302	0.3292	0.2403	0.2738	0.0732	0.2381	0.204
BCC	0.0842	0.1019	0.1055	0.0926	0.0998	0.1295	0.1587	0.1006	0.0925
A15 (β-W)	0.0103	0.0599	0.0131	0.0165	0.074	0.0834	0.1855	0.0781	0.0749
HCP	0.0237	0.0276	0.0278	0.0216	0.0244	0.0301	0.0401	0.0312	0.0293
9R	0.0179	0.0186	0.0205	0.0161	0.0168	0.0204	0.0271	0.0211	0.0181
DHCP	0.0152	0.0141	0.0161	0.0133	0.0128	0.0154	0.0204	0.0161	0.0104
FCC	0	0	0	0	0	0	0	0	0

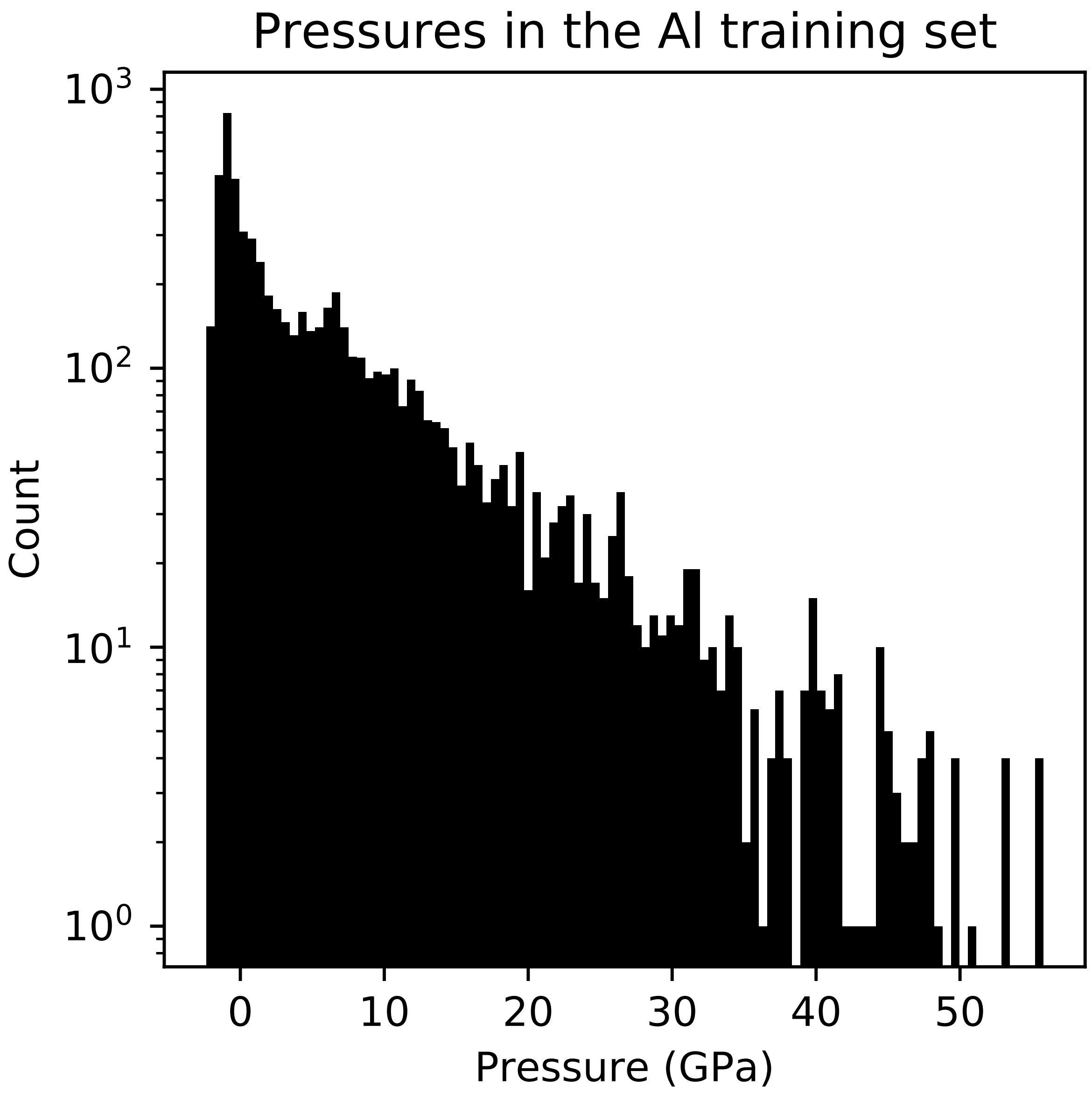

Supplementary Figure 1: Distribution of pressures in the training data set.

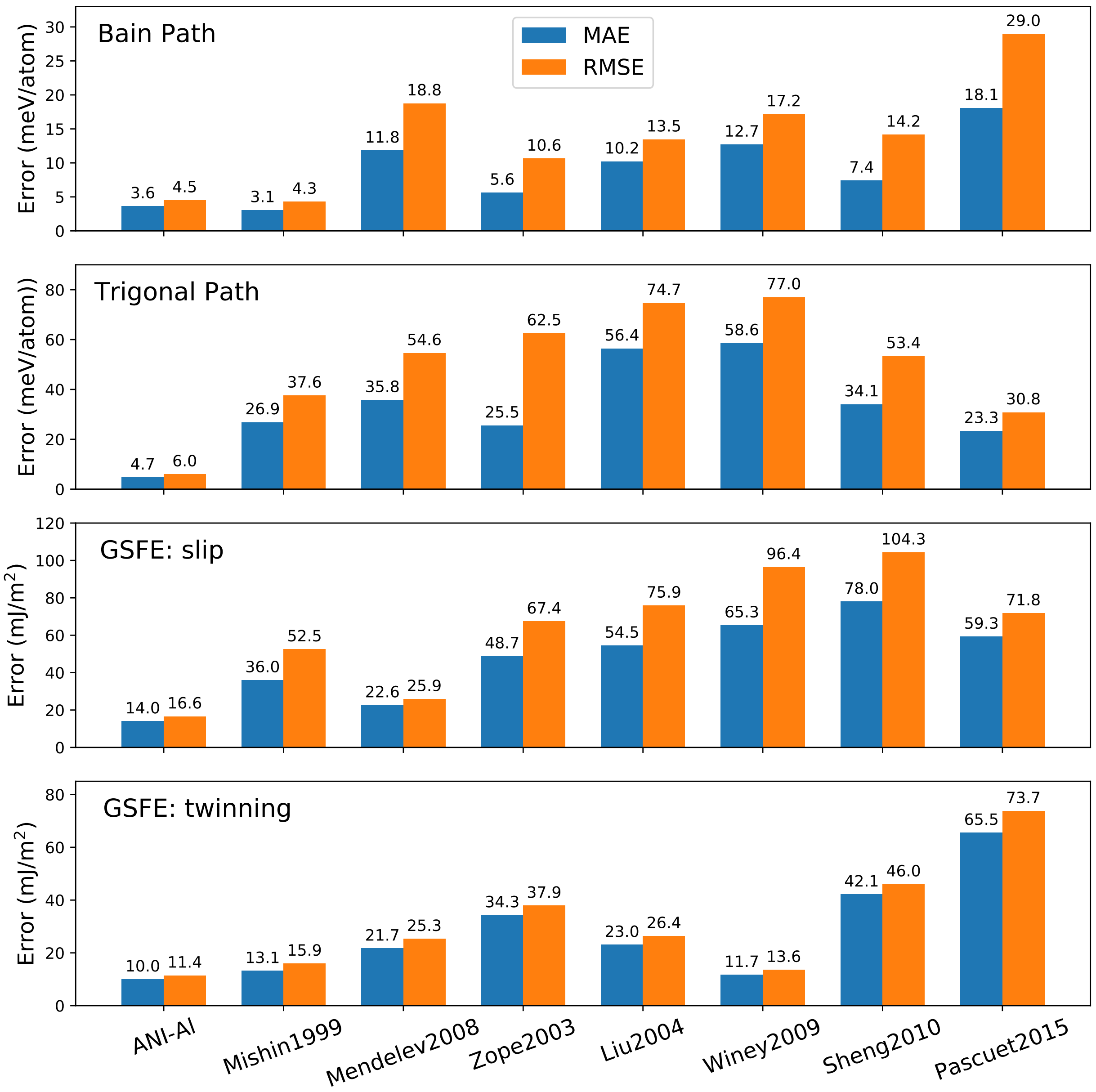

Supplementary Figure 2: Complete path errors for each method compared to reference DFT data. The methods are order from left to right by the lowest average error.

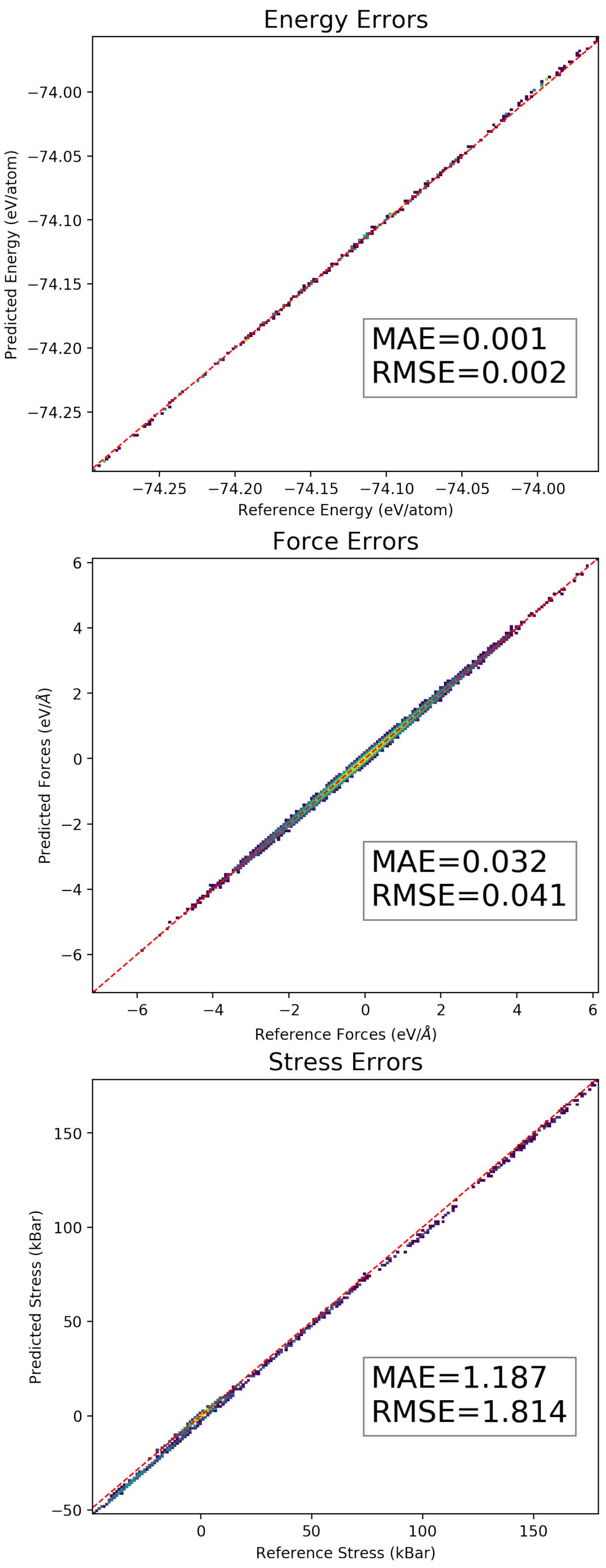

Supplementary Figure 3: Correlation plots of energy, forces, and pressures for liquid Al MD trajectories over a grid of atomic volumes (0.8Å^3 to 1.2Å^3) and temperatures (1000K to 2000K).

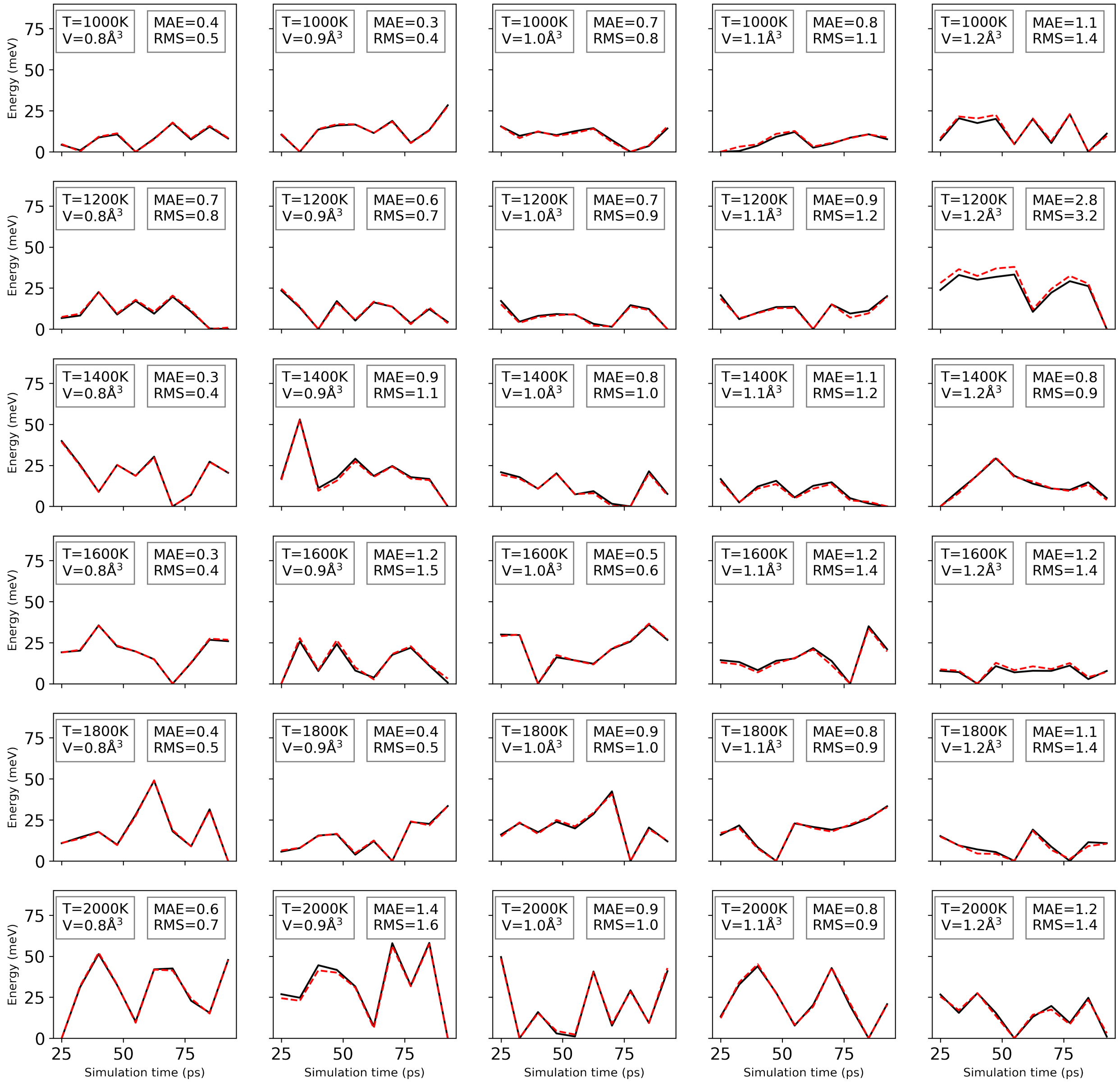

Supplementary Figure 4: Plots of liquid Al MD trajectories over a grid of atomic volumes (0.8Å^3 to 1.2Å^3) and temperatures (1000K to 2000K).

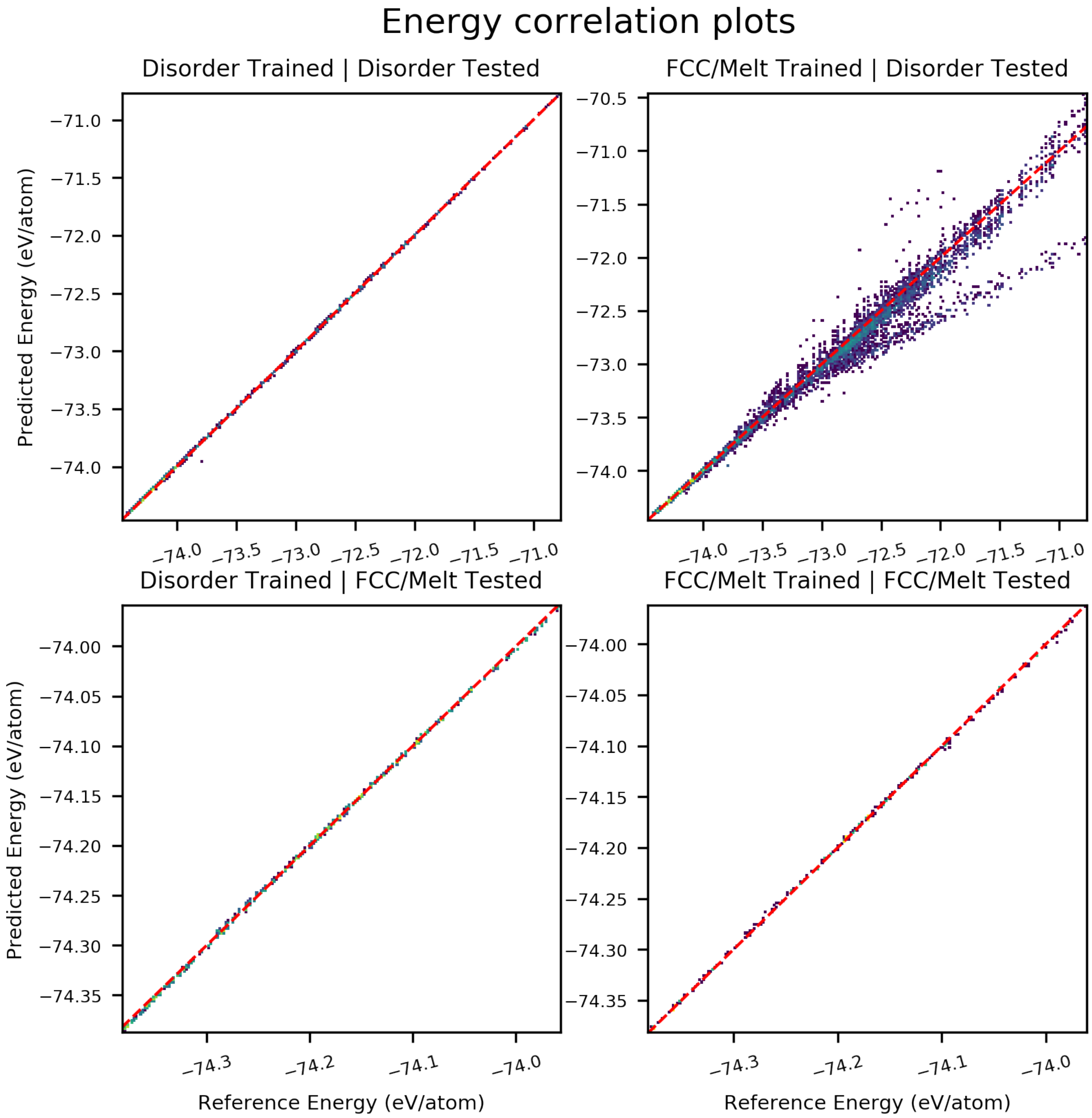

Supplementary Figure 5: Energy correlations. “FCC/Melt” refers to the dataset collected near equilibrium for these two phases. “Disorder” refers to the dataset collected automatically by the active learning procedure (the AL dataset of the main text), and includes highly out of equilibrium data.

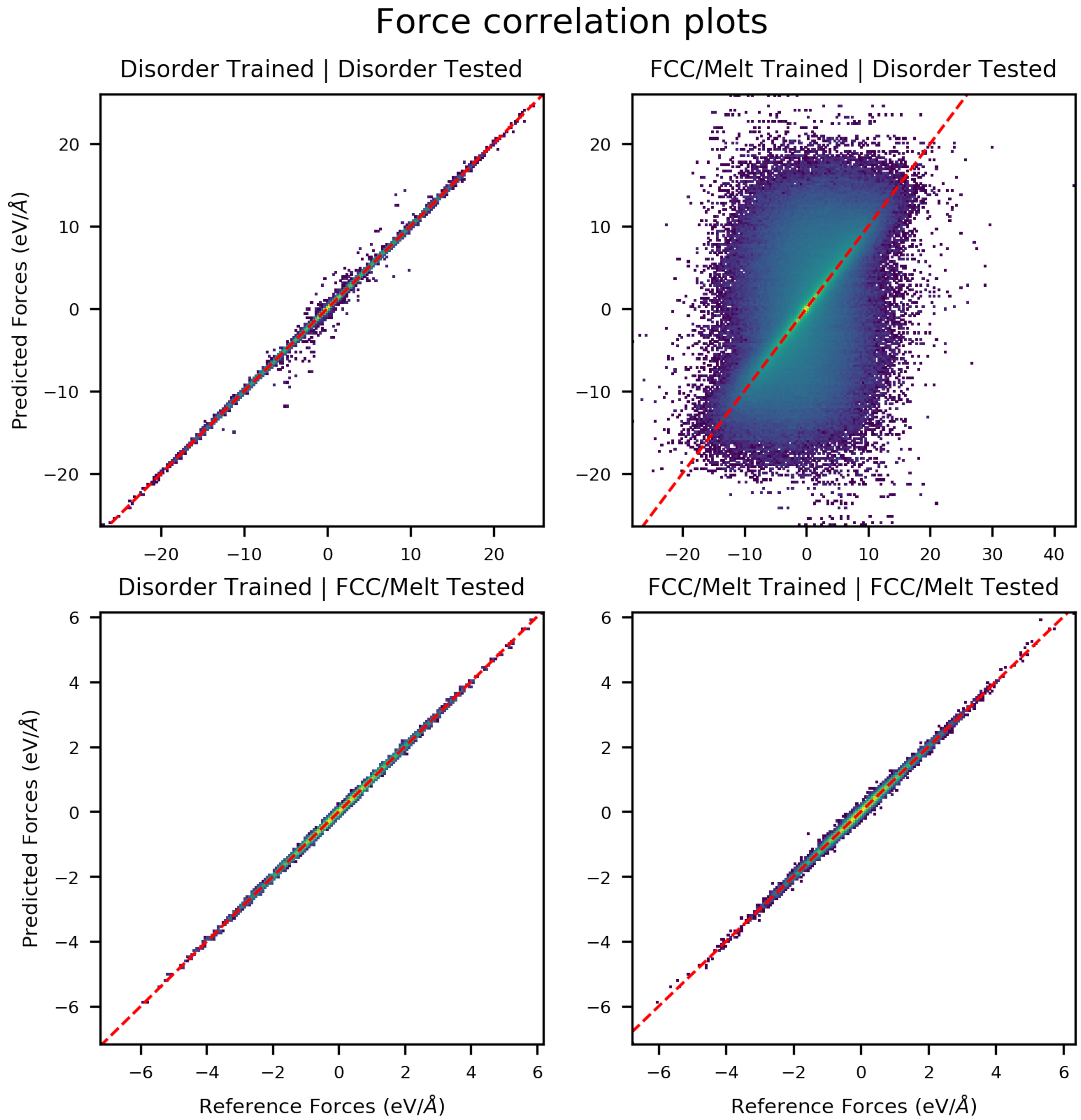

Supplementary Figure 6: Force component correlations.

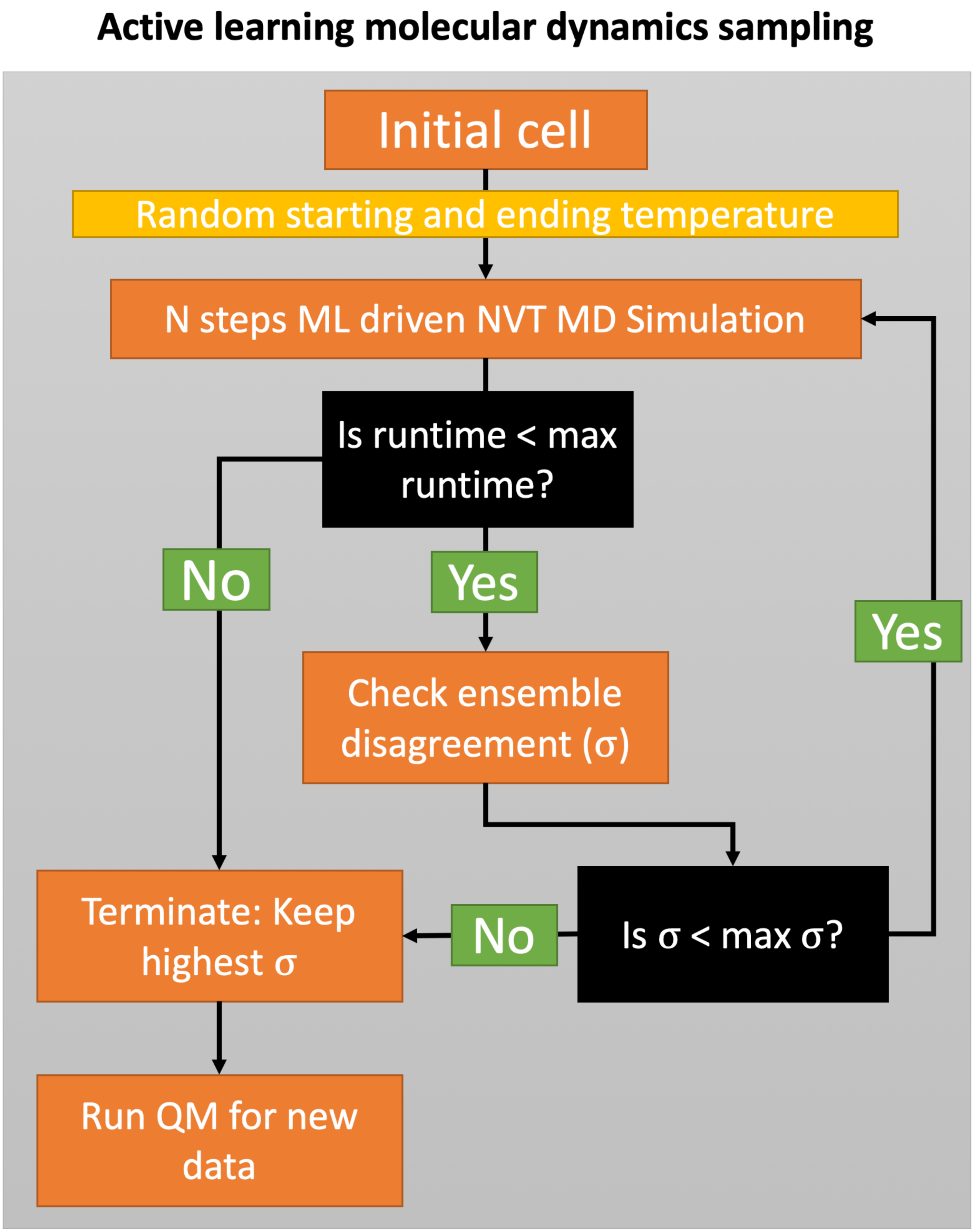

Supplementary Figure 7: Diagram of the active learning algorithm.

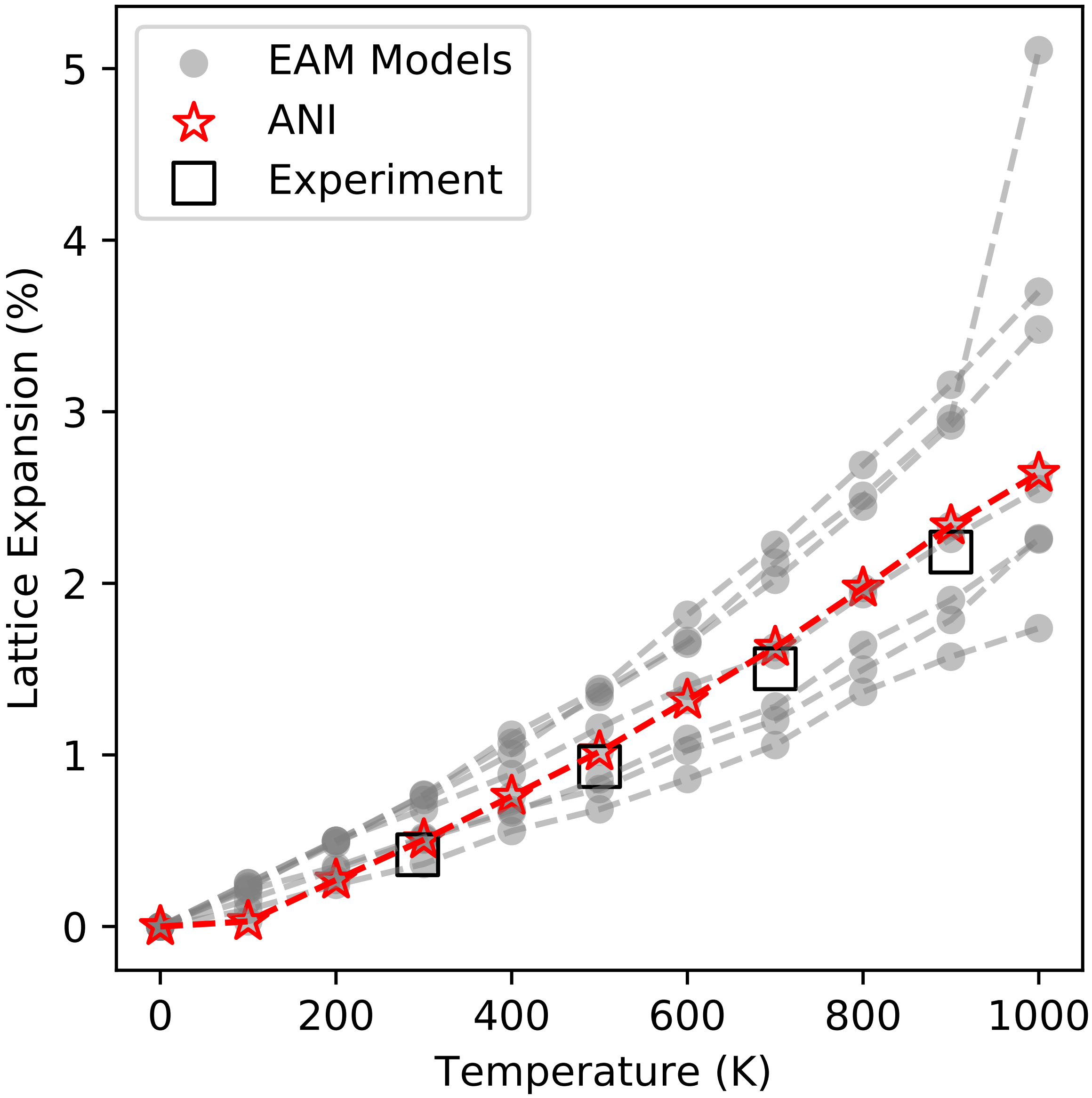

Supplementary Figure 8: Thermal expansion of ANI-Al compared to experiment and other EAM models.

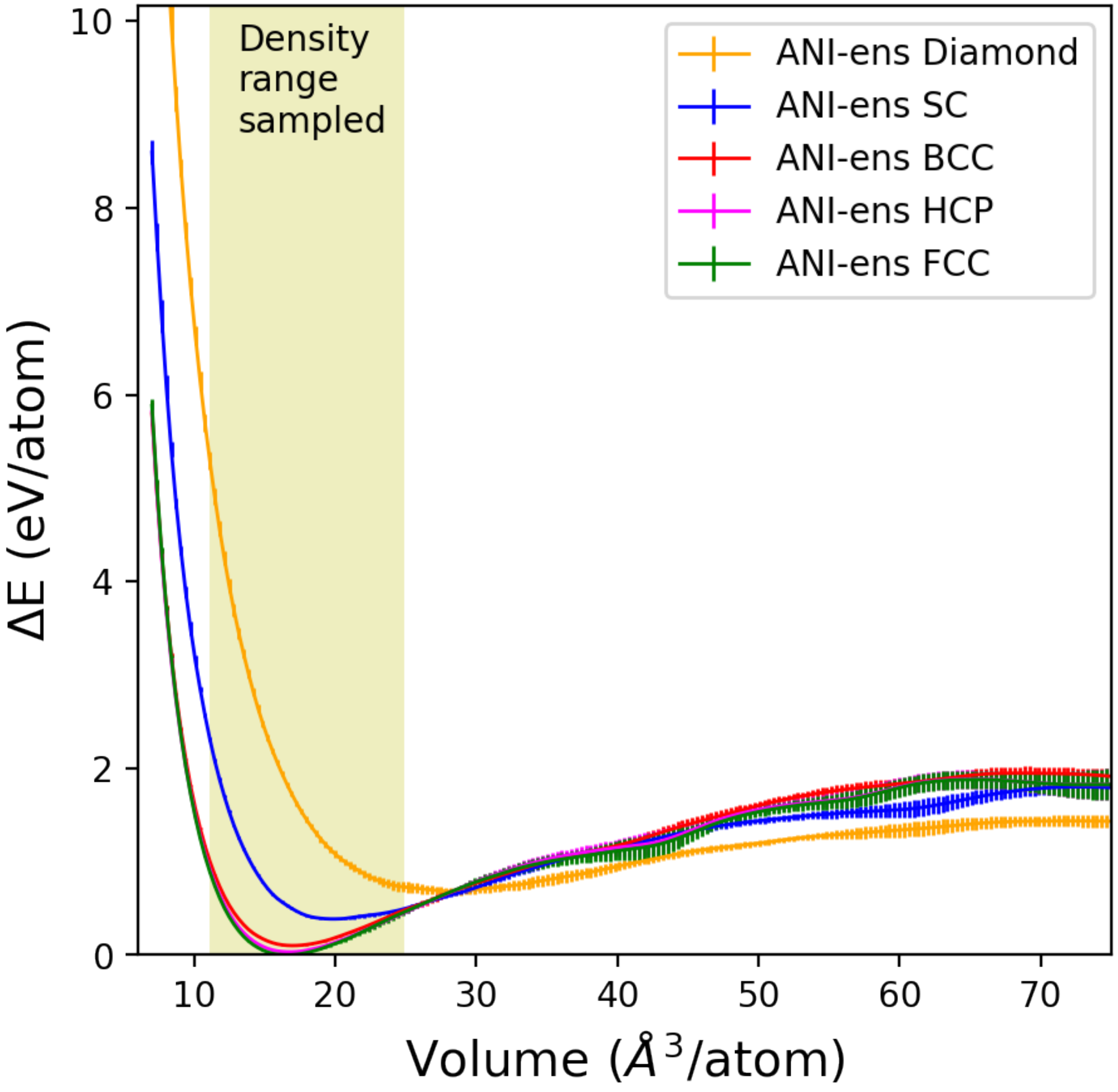

Supplementary Figure 9: Extended cold curves for ANI-Al ensemble. At zero temperature ANI-Al predicts an FCC-to-HCP transition at 154.8 GPa, and an HCP-to-BCC transition at 392.7 GPa. The BCC crystal becomes lower energy than FCC above 261.5 GPa.

References

[1] P. Giannozzi, S. Baroni, N. Bonini, M. Calandra, R. Car, C. Cavazzoni, D. Ceresoli, G. L. Chiarotti, M. Cococcioni, I. Dabo, A. Dal Corso, S. De Gironcoli, S. Fabris, G. Fratesi, R. Gebauer, U. Gerstmann, C. Gougoussis, A. Kokalj, M. Lazzeri, L. Martin-Samos, N. Marzari, F. Mauri, R. Mazzarello, S. Paolini, A. Pasquarello, L. Paulatto, C. Sbraccia, S. Scandolo, G. Sclauzero, A. P. Seitsonen, A. Smogunov, P. Umari, and R. M. Wentzcovitch, "QUAN-

TUM ESPRESSO: A modular and open-source software project for quantum simulations of materials," *Journal of Physics Condensed Matter*, vol. 21, p. 395502, 9 2009.

[2] P. Giannozzi, O. Andreussi, T. Brumme, O. Bunau, M. Buongiorno Nardelli, M. Calandra, R. Car, C. Cavazzoni, D. Ceresoli, M. Cococcioni, N. Colonna, I. Carnimeo, A. Dal Corso, S. De Gironcoli, P. Delugas, R. A. Distasio, A. Ferretti, A. Floris, G. Fratesi, G. Fugallo, R. Gebauer, U. Gerstmann, F. Giustino, T. Gorni, J. Jia, M. Kawamura, H. Y. Ko, A. Kokalj, E. Kücükbenli, M. Lazzeri, M. Marsili, N. Marzari, F. Mauri, N. L. Nguyen, H. V. Nguyen, A. Otero-De-La-Roza, L. Paulatto, S. Poncé, D. Rocca, R. Sabatini, B. Santra, M. Schlipf, A. P. Seitsonen, A. Smogunov, I. Timrov, T. Thonhauser, P. Umari, N. Vast, X. Wu, and S. Baroni, "Advanced capabilities for materials modelling with Quantum ESPRESSO," *Journal of Physics Condensed Matter*, vol. 29, no. 46, 2017.

[3] N. Marzari, D. Vanderbilt, A. De Vita, and M. C. Payne, "Thermal contraction and disordering of the al(110) surface," *Physical Review Letters*, vol. 82, pp. 3296–3299, 4 1999.

[4] J. S. Smith, B. Nebgen, N. Lubbers, O. Isayev, and A. E. Roitberg, "Less is more: sampling chemical space with active learning," *The Journal of Chemical Physics*, vol. 148, p. 241733, 6 2018.

[5] J. S. Smith, O. Isayev, and A. E. Roitberg, "ANI-1: an extensible neural network potential with DFT accuracy at force field computational cost," *Chem. Sci.*, vol. 8, no. 4, pp. 3192–3203, 2017.

[6] D. Kingma and J. Ba, "Adam: A Method for Stochastic Optimization," *arXiv*, p. Preprint at https://arxiv.org/abs/1412.6980, 12 2014.

[7] K. Yao, J. E. Herr, D. W. Toth, R. Mcintyre, and J. Parkhill, "The TensorMol-0.1 Model Chemistry: a Neural Network Augmented with Long-Range Physics," *Chemical Science*, vol. 9, pp. 2261–2269, 2 2017.

[8] N. Lubbers, J. S. Smith, and K. Barros, "Hierarchical modeling of molecular energies using a deep neural network," *The Journal of Chemical Physics*, vol. 148, p. 241715, 6 2018.

[9] L. Zhang, J. Han, H. Wang, R. Car, and E. Weinan, "Deep Potential Molecular Dynamics: A Scalable Model with the Accuracy of Quantum Mechanics," *Physical Review Letters*, vol. 120, no. 14, 2018.

[10] NeuroChem binaries available online at https://github.com/atomistic-ml/neurochem.

[11] J. S. Smith, N. Lubbers, A. P. Thompson, and K. Barros, "Simple and efficient algorithms for training machine learning potentials to force data," p. Preprint at: https://arxiv.org/abs/2006.05475, 6 2020.

[12] P. G. Poličar, M. Stražar, and B. Zupan, "OpenTSNE: A modular Python library for t-SNE dimensionality reduction and embedding," *bioRxiv*, p. Preprint at: https://doi.org/10.1101/731877, 2019.

[13] J. R. Morris, C. Z. Wang, K. M. Ho, and C. T. Chan, "Melting line of aluminum from simulations of coexisting phases," *Physical Review B*, vol. 49, pp. 3109–3115, 2 1994.

[14] J. R. Morris and X. Song, "The melting lines of model systems calculated from coexistence simulations," *Journal of Chemical Physics*, vol. 116, pp. 9352–9358, 6 2002.

[15] J. R. Espinosa, E. Sanz, C. Valeriani, and C. Vega, "On fluid-solid direct coexistence simulations: The pseudo-hard sphere model," *Journal of Chemical Physics*, vol. 139, p. 144502, 10 2013.

[16] M. Mendelev, M. Kramer, C. Becker, and M. Asta, "Analysis of semi-empirical interatomic potentials appropriate for simulation of crystalline and liquid Al and Cu," *Philosophical Magazine*, vol. 88, pp. 1723–1750, 4 2008.

[17] W. P. Davey, "Precision measurements of the lattice constants of twelve common metals," *Physical Review*, vol. 25, pp. 753–761, 6 1925.

[18] G. Simmons and H. Wang, *Single Crystal Elastic Constants and Calculated Aggregate Properties. A Handbook.* Cambridge, Mass: M.I.T. Press, 1971.

[19] H. E. Schaefer, R. Gugelmeier, M. Schmolz, and A. Seeger, "Positron Lifetime Spectroscopy and Trapping at Vacancies in Aluminium," *Materials Science Forum*, vol. 15-18, pp. 111–116, 1 1987.

[20] M. J. Fluss, L. C. Smedskjaer, M. K. Chason, D. G. Legnini, and R. W. Siegel, "Measurements of the vacancy formation enthalpy in aluminum using positron annihilation spectroscopy," *Physical Review B*, vol. 17, pp. 3444–3455, 5 1978.

[21] I. F. Bainbridge and J. A. Taylor, "The surface tension of pure aluminum and aluminum alloys," *Metallurgical and Materials Transactions A: Physical Metallurgy and Materials Science*, vol. 44, pp. 3901–3909, 8 2013.

[22] Von L. E. Murr, "Interfacial Phenomena in Metal and Alloys.," *Physik in unserer Zeit*, vol. 8, no. 1, pp. 30–30, 1977.

[23] J. H. Petersen, A. Mikkelsen, M. M. Nielsen, and D. L. Adams, "Structure of Al(100)-c(2 x 2)-Li: A binary surface alloy," *Physical Review B - Condensed Matter and Materials Physics*, vol. 60, pp. 5963–5968, 8 1999.

[24] J. R. Noonan and H. L. Davis, "Truncation-induced multilayer relaxation of the A1(110) surface," *Physical Review B*, vol. 29, pp. 4349–4355, 4 1984.

[25] D. L. Adams, H. B. Nielsen, and J. N. Andersen, "Quantitative Analysis of LEED Measurements," *Physica Scripta*, vol. 1983, pp. 22–28, 1 1983.

[26] R. H. Rautioaho, "An Interatomic Pair Potential for Aluminium Calculation of Stacking Fault Energy," *Physica Status Solidi (B)*, vol. 112, pp. 83–89, 7 1982.

[27] "Thermochemical data," in *Smithells Metals Reference Book*, pp. 1–58, Elsevier, 2003.

[28] G. P. Pun, R. Batra, R. Ramprasad, and Y. Mishin, "Physically informed artificial neural networks for atomistic modeling of materials," *Nature Communications*, vol. 10, p. 2339, 12 2019.

[29] L. Zhang, D. Y. Lin, H. Wang, R. Car, and E. Weinan, "Active learning of uniformly accurate interatomic potentials for materials simulation," *Physical Review Materials*, vol. 3, p. 023804, 2 2019.
